# Ketamine Across the Dose–State Continuum: EEG Signatures, Network Dynamics, and Implications for Brain-State Monitoring in Anesthesia and Critical Care

**DOI:** 10.3390/brainsci16080845

**Published:** 2026-08-09

**Authors:** Vikas Chauhan, Fareena Khan

**Affiliations:** Department of Anesthesiology, University of Mississippi Medical Center, Jackson, MS 39216, USA

**Keywords:** ketamine, electroencephalography, depth of anesthesia, brain connectivity, dissociation, consciousness, critical care, antidepressant

## Abstract

**Highlights:**

**What are the main findings?**

- Ketamine produces a dose-dependent continuum of brain states whose EEG signatures are non-suppressive, fast, high-frequency activity and a gamma-burst pattern, fundamentally unlike the progressive slowing of GABAergic anesthetics.
- Processed depth indices may remain paradoxically elevated because ketamine disrupts network integration without uniform cortical suppression.

**What are the implications of the main findings?**

- Ketamine-associated brain states should be interpreted using raw EEG, spectrograms, dosing, co-administered hypnotics, and clinical signs rather than a scalar index alone.
- Connectivity, complexity, and spectral-slope measures are promising monitoring biomarkers but require prospective clinical validation.

**Abstract:**

Ketamine produces clinical states ranging from subanesthetic analgesia and dissociation to anesthetic-dose behavioral unresponsiveness. Its electroencephalographic (EEG) effects differ from the slow-delta and frontal-alpha patterns commonly observed with GABAergic-dominant anesthetics and vary with exposure, administration kinetics, and co-administered agents. After anesthetic bolus dosing, ketamine may produce alternating slow-delta and gamma activity; at lower exposures, spectral and connectivity findings are more heterogeneous. When ketamine is added to propofol or volatile anesthesia, bispectral index and spectral-entropy values may remain elevated or increase, limiting their interpretation as stand-alone measures of hypnotic state. Prior syntheses have largely addressed molecular, cellular, and cortical-circuit mechanisms of dissociation; this narrative review instead synthesizes human EEG, connectivity, imaging, and selected mechanistic evidence using a dose–state framework. We distinguish behavioral responsiveness, environmental connectedness, and conscious experience; assess which network measures are technically derivable from clinical scalp recordings; and consider implications for operating-room and critical-care monitoring. Available evidence supports a cautious interpretation of processed indices and greater attention to the raw EEG, spectrogram, background hypnotic, administration pattern, and clinical context. Validated ketamine-specific EEG biomarkers and prospective monitoring algorithms are not yet available. Potential links between acute EEG effects and antidepressant mechanisms are discussed as shared upstream pathways rather than a single electrophysiological state.

## 1. Introduction

Ketamine has a distinctive position in anesthetic pharmacology. Introduced in the 1960s and originally characterized as a “dissociative anesthetic”, it acts principally through non-competitive antagonism of the N-methyl-D-aspartate (NMDA) receptor, a mechanism distinct from the γ-aminobutyric acid (GABA)-ergic potentiation that underlies most intravenous and volatile anesthetics [1,2]. Its clinical effects are strikingly dose-dependent: subanesthetic doses produce analgesia and a dissociative state marked by perceptual detachment and altered sensory experience, whereas higher doses induce a state of unresponsiveness suitable for general anesthesia. Equally distinctive is its physiological profile—relative preservation of spontaneous ventilation and sympathetic tone compared with many conventional hypnotics, together with bronchodilation—which has long made it valuable in patients for whom hemodynamic or respiratory depression is a particular concern. Ketamine thus spans, within a single drug, a continuum of states from light sedation through dissociation to surgical anesthesia.

This versatility underlies a marked expansion of ketamine’s role across perioperative and critical-care medicine. As a component of multimodal, opioid-sparing analgesia, it is now widely incorporated into perioperative pain regimens, and its hemodynamic and respiratory profile continues to recommend it for induction in trauma, shock, and other states of cardiovascular instability [3]. In the intensive-care unit, it is increasingly used for analgesia-based sedation and is an established option in the management of refractory and super-refractory status epilepticus, while in emergency and procedural settings it remains a mainstay of dissociative sedation. This clinical resurgence has been amplified by ketamine’s emergence as a rapid-acting antidepressant and the regulatory approval of its S-enantiomer for treatment-resistant depression, developments that have substantially increased both research attention and patient exposure [4]. Clinicians now encounter ketamine across its full dose range, frequently co-administered with GABAergic agents, more often than at any point in its history.

Over the same period, monitoring of the anesthetized and sedated brain has itself moved toward routine electrophysiology. Processed electroencephalographic (EEG) indices intended to track the hypnotic component of anesthesia are now in common intraoperative use, typically targeted to defined ranges to avoid both intraoperative awareness and unnecessarily deep anesthesia [5,6], and raw and quantitative EEG, including continuous EEG, is increasingly applied at the bedside in critical care [7]. These monitors and their interpretive heuristics were, however, developed and validated largely with GABAergic anesthetics, whose effect on the EEG is an orderly, dose-dependent progression toward slowing and, at sufficient depth, burst suppression [8,9]. Ketamine is an important exception to this pattern.

Recent reviews have primarily emphasized ketamine’s molecular, cellular, and cortical-circuit mechanisms, including N-methyl-D-aspartate receptor antagonism, interneuron disinhibition, altered thalamocortical signaling, and the layer- and region-specific dynamics associated with dissociation [10]. Less clearly synthesized is how ketamine-related electroencephalographic and network changes should be interpreted at the bedside. Because many processed EEG indices were developed and validated predominantly with GABAergic-dominant anesthetics, ketamine-associated fast activity may produce an index–state discordance: processed-index values may remain elevated or increase without indicating inadequate hypnosis, particularly when ketamine is used as an adjunct. Such changes may prompt unnecessary escalation of the co-administered hypnotic or volatile anesthetic [11,12]. In neurocritical care, ketamine-related EEG changes may also complicate assessment of arousal and ictal activity; validated criteria for distinguishing drug-related fast activity from clinically meaningful EEG evolution remain lacking [7,13].

Evidence is uneven across ketamine-associated states. The best-described anesthetic-dose pattern is an alternating slow-delta and gamma “gamma-burst” signature following bolus induction [8,14], whereas subanesthetic and dissociative exposures have yielded more heterogeneous spectral and connectivity findings [15,16]. Much of the large-scale network evidence derives from functional imaging in awake volunteers or subanesthetic conditions, and its generalizability to operating-room and intensive-care populations remains uncertain [17,18]. Findings are additionally influenced by dose, administration pattern, and co-administered anesthetics [12]. We therefore use a dose–state framework as an organizing heuristic—not a validated depth scale—to synthesize ketamine-associated EEG and network signatures from subanesthetic exposure through dissociation, anesthetic unresponsiveness, and emergence, while distinguishing clinically supported inferences from unresolved questions.

### Review Approach and Literature Identification

This is a narrative review rather than a systematic one. It concentrates on macroscale, scalp-accessible EEG and large-scale network signatures of ketamine across the dose–state continuum and on how those signatures should be interpreted for perioperative and critical-care brain-state monitoring. It does not attempt an exhaustive survey of every report, nor a detailed account of molecular pharmacology or non-EEG neuroimaging except where these bear on bedside interpretation.

The contribution of this review is a clinical reframing rather than a discovery, and it is worth situating against the adjacent literature. Recent syntheses address related but distinct aspects of ketamine neurophysiology: one emphasizes the cellular, regional, and cortical-circuit mechanisms contributing to dissociation [10], while two recent systematic reviews synthesize electrophysiological findings in healthy and depressed populations—the first covering EEG and magnetoencephalographic spectral signatures of ketamine alongside psilocybin and lysergic acid diethylamide and the second covering magnetoencephalographic oscillatory and connectivity changes after subanesthetic ketamine in healthy individuals and in patients with major depressive disorder or treatment-resistant depression [19,20]. Neither organizes the evidence across subanesthetic, dissociative, anesthetic-dose, and recovery states, and neither centers processed-index discordance, administration pattern, co-administered hypnotics, or bedside interpretation because neither is directed at perioperative or critical-care monitoring. The present review therefore offers a dose–state, monitoring-oriented synthesis of human EEG, connectivity, imaging, and selected mechanistic evidence rather than a claim of new physiology.

This narrative review was informed by targeted searches of PubMed/MEDLINE, last updated in July 2026, supplemented by backward citation searching of included studies and recent reviews and targeted searches of reference lists and journal websites. Search concepts combined “ketamine” or “esketamine” with “electroencephalography,” “EEG,” “bispectral index,” “spectral entropy,” “spectrogram,” “connectivity,” “coherence,” “complexity,” “consciousness,” “dissociation,” “anesthesia,” “sedation,” and “critical care.” Human clinical and neurophysiological studies were prioritized; preclinical studies were included when they clarified mechanisms not directly accessible in humans. Because the objective was an interpretive narrative synthesis rather than exhaustive study identification or pooled effect estimation, formal duplicate screening, risk-of-bias assessment, and meta-analysis were not performed, and no review protocol was registered. No date restriction was applied; records indexed from database inception through July 2026 were considered. Study limitations were nevertheless weighed narratively across design, sample size, selection, blinding, confounding, outcome measurement, directness, and clinical applicability, and these judgments are summarized in the evidence table in Section 9.

## 2. Pharmacological and Circuit Substrate

Ketamine’s macroscale signatures rest on a molecular and circuit-level account reviewed in detail elsewhere [10]; we summarize only what is needed to make the later electrophysiology interpretable. The drug’s primary action is non-competitive, open-channel antagonism of the NMDA glutamate receptor, but its cortical effect is not uniform inhibition. One influential model proposes that, at lower concentrations, ketamine preferentially reduces NMDA-receptor signaling on fast-spiking GABAergic interneurons, which are tonically active and especially sensitive, so that disinhibition of pyramidal neurons increases cortical excitability and glutamate release [21]. This glutamate surge drives transmission through AMPA receptors, and enhanced AMPA-mediated thalamocortical and corticocortical throughput has been proposed as a proximate contributor to ketamine-associated gamma activity and to processed-index elevation [22]. These remain mechanistic models rather than direct explanations of any particular bedside index. AMPA-mediated signaling is also implicated in the downstream plasticity associated with ketamine’s rapid antidepressant action (Section 8), which suggests a shared synaptic origin rather than an established common pathway.

Two further mechanisms shape the anesthetic state. Ketamine inhibits hyperpolarization-activated cyclic nucleotide-gated (HCN1) channels, a contribution demonstrated by the reduced hypnotic sensitivity of HCN1-deficient animals, which helps explain how a locally excitatory drug can nonetheless produce unresponsiveness [23]. Beyond these channel- and receptor-level actions, ketamine may also engage intracellular targets—accumulating in acidic organelles and acting through synaptic trapping to relieve NMDA-receptor hypofunction—mechanisms that extend its pharmacology beyond surface NMDA blockade [24]. At the network level its actions converge on the thalamocortical system, where altered effective connectivity accompanies the transition to unresponsiveness even as thalamic activity is comparatively preserved relative to GABAergic agents (Section 5). The essential feature to carry forward is that ketamine does not silence the cortex; it disinhibits and reorganizes it, so the brain it produces is active, high-frequency, and metabolically engaged rather than suppressed. Recent preclinical work implicates retrosplenial cortex parvalbumin interneurons as a key circuit substrate for dissociation-related behaviors and ketamine-associated slow oscillatory activity, supporting a model in which dissociation emerges from region- and cell-type-specific disruption rather than uniform cortical excitation [25].

## 3. The Ketamine Dose–State Continuum

A single drug produces a graded spectrum of states as concentration rises, and this review is organized around that spectrum. It is worth stating at the outset what the continuum is and is not. It is a heuristic for arranging an uneven literature—a way of relating doses, behavioral states, and brain signatures within a common frame—rather than a validated mapping in which each dose corresponds to a fixed signature. The boundaries between ranges are approximate and overlapping; the doses producing a given state vary substantially between individuals and with the pattern and rate of administration, and the evidence supporting each range is uneven, as later sections make clear. Importantly, the dose–signature relationships described here are drawn largely from bolus administration, and the most distinctive patterns—such as the anesthetic-range gamma-burst—have been best characterized after bolus administration; a continuous infusion delivering the same average exposure may produce a different EEG signature, a caveat developed below. With those caveats, four broad regions can be distinguished, summarized in Table 1.

At the lowest, subanesthetic end—on the order of 0.1–0.5 mg/kg intravenously, or comparable low-dose infusions—ketamine produces analgesia and the mild perceptual and cognitive alterations exploited for opioid-sparing pain control [3] and, at around 0.5 mg/kg by slow infusion, for rapid antidepressant effect [26,27]. As the concentration increases (roughly 0.5–1 mg/kg), perceptual detachment deepens into frank dissociation—the detachment from body, environment, and self that gives the drug its defining phenomenology—in the range used for dissociative procedural sedation [28]. Higher still (approximately 1–2 mg/kg as an induction dose), ketamine produces an unresponsive state suitable for general anesthesia [29]. Emergence and recovery then constitute a distinct phase rather than a simple reversal, marked clinically by the dreams, hallucinations, and emergence phenomena that follow ketamine anesthesia [30] and, experimentally, by the drug’s capacity to alter the trajectory of recovery from co-administered anesthetics [31]. These figures are indicative, offered to orient the reader rather than as thresholds. Recent work further suggests that ketamine’s subjective dose–state continuum may be accompanied by progressive stabilization of cortical dynamics, particularly in higher-frequency ranges, providing a dynamical-systems complement to spectral and connectivity-based EEG interpretations [32]. Cross-anesthetic imaging studies further suggest that dose escalation may produce multiple paths away from wake-like critical dynamics, with ketamine showing distinct dose-dependent network modes compared with GABAergic or volatile anesthetics [33].

Two pharmacological qualifications cut across the continuum. The first is the distinction between racemic ketamine and its S-enantiomer (esketamine), which differ in potency and receptor affinity and now predominate in different settings—esketamine in much psychiatric and some European anesthetic practice, the racemate elsewhere. Where their EEG and network signatures have been compared the differences appear modest relative to the dose-related changes that organize this review, but direct comparisons are few. The second qualification is more consequential for interpreting the literature: the same total dose produces different effects depending on whether it is delivered as a bolus or a continuous infusion. Boluses generate rapid, high peak concentrations and the abrupt transitions captured in much of the anesthetic-signature work, whereas infusions produce the steadier states used for analgesia, sedation, and seizure control. Because the anesthetic ranges of the continuum have been studied largely with boluses and the subanesthetic ranges largely with infusions, the dose and administration pattern are partly confounded across the evidence, and processed-index behavior in particular differs between the two. This confound recurs throughout the sections that follow and is taken up as a research priority in Section 9; we raise it here because it qualifies every claim that maps a dose to a signature.

## 4. Electroencephalographic Signatures Across the Dose–State Continuum

The electroencephalographic terms used in this section and in those that follow, with the computation each denotes and its approximate frequency range, are defined in Table 2.

**Figure 1:**
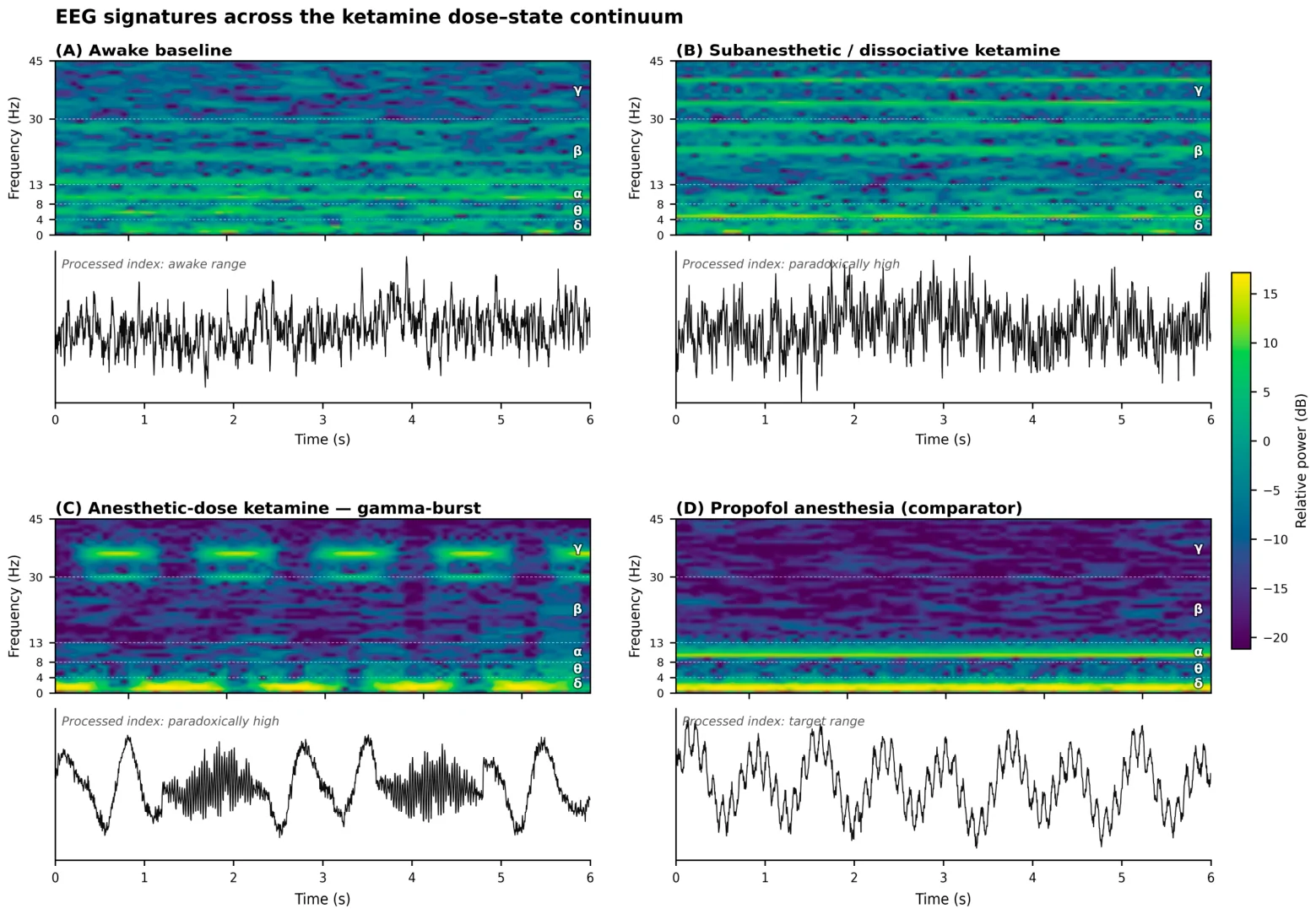
EEG signatures across the ketamine dose–state continuum. Schematic frontal EEG traces and spectrograms illustrate qualitative differences between awake baseline, subanesthetic/dissociative ketamine, anesthetic-dose ketamine, and propofol anesthesia. Subanesthetic ketamine may be associated with increased fast activity and processed-index values that remain elevated or rise, whereas anesthetic-dose ketamine may show alternating slow-delta and gamma activity despite behavioral unresponsiveness. Propofol is shown as a GABAergic comparator with slow-delta and coherent alpha activity. Approximate conventional frequency bands are marked on each spectrogram by dashed lines and labeled δ (0.5–4 Hz), θ (4–8 Hz), α (8–13 Hz), β (13–30 Hz), and γ (30–45 Hz); boundaries are conventional approximations and vary between studies. Traces and spectrograms are schematic and do not represent patient-specific recordings or commercial monitor output. Abbreviations: dB, decibels; EEG, electroencephalography; GABA, gamma-aminobutyric acid; Hz, hertz; s, seconds.

Of ketamine’s brain-state correlates, its scalp EEG signatures are both the best characterized and the most relevant to the bedside—but they are not described evenly across the dose range. The signature of anesthetic-dose ketamine has been detailed in humans, whereas the analgesic and dissociative ranges are sketched more partially, and the oscillatory features that would distinguish a sedative state from a non-gamma-burst anesthetic state have not been formally established [9]. We therefore begin at the anesthetic end, where the evidence is firmest, before turning to the lower-dose ranges and to the cross-cutting properties—spectral slope and signal complexity—that most sharply separate ketamine from the GABAergic agents around which depth monitoring was designed. Figure 1 summarizes the signatures schematically alongside a propofol comparator.

### 4.1. The Anesthetic-Dose Signature: The Gamma-Burst Pattern

The best-described anesthetic-dose pattern is the “gamma-burst.” Following an induction bolus, the frontal EEG develops a rhythmic alternation between slow-delta oscillations (≈0.1–4 Hz) and bursts of gamma activity (≈27–40 Hz), accompanied by increased theta (≈4–8 Hz) and reduced alpha and beta (≈10–24 Hz) power [14]. Over the following minutes the rhythmic alternation tends to give way to a more sustained, less periodic gamma pattern [14]. These dynamics are not peculiar to scalp recording: a comparable structure of high-power gamma alternating with slow-delta activity is evident in non-human primate local field potentials and has been captured statistically—as a sequence of discrete spectral states identified by a hidden Markov model—across both macaque and human signals [34]. It is worth emphasizing that this pattern is, in the first instance, a bolus phenomenon: it has been characterized following induction boluses and reflects the rapid, high peak concentration these produce, so an equivalent average exposure delivered by slow infusion need not generate the same florid slow-gamma alternation, and the signature is partly one of administration kinetics rather than of dose alone. It has been proposed that the gamma-burst pattern is the ketamine analogue of GABAergic burst suppression, emerging at higher effect-site concentrations [9]. The relationship to anesthetic depth is not one-to-one; however, unresponsiveness to salient stimulation persists after the gamma-burst pattern dissipates, at which point the EEG shows increased theta and gamma with decreased alpha–beta power [9,14]. Under the bolus conditions studied, the pattern therefore supports a ketamine-associated effect state and a particular phase of its action more reliably than it grades depth in the way clinicians expect from GABAergic agents.

### 4.2. The Subanesthetic and Dissociative Range

At the lower doses used for analgesia and to elicit dissociation, the picture is both different and less settled. The most consistently reported scalp finding is a reduction in alpha (≈8–13 Hz) and low-beta (≈13–20 Hz) oscillation power [15,35,36]. Changes in theta are less consistent and appear to depend on administration: bolus dosing has been associated with increased frontal theta, whereas slow subanesthetic infusion has been associated with reduced theta, and experimental work has long shown that ketamine modulates both theta and gamma rhythms [37]. What is largely missing is a validated mapping from these features to the subjective state: the same decrease in alpha and low-beta power has been linked both to the dissociative experience and to ketamine given as an anesthetic adjunct, so the surface oscillations appear to track drug effect and arousal more than dissociation as such [35]. Several of the more striking correlates of the dissociative range—a posteromedial rhythm near 3 Hz, frequency-specific engagement of prefrontal and hippocampal cortex, and reorganization of large-scale networks—have been demonstrated only with intracranial recording or functional imaging [38] and are not recoverable from a routine frontal montage, a point developed in Section 5.

### 4.3. A Non-Suppressive Cortex: Spectral Slope and Complexity

Cutting across the dose range is a property that distinguishes ketamine from GABAergic anesthetics: its cortical activity does not collapse toward the low-complexity, suppression-dominated regime those agents produce. The aperiodic slope of the EEG power spectrum, which steepens markedly under propofol and xenon as consciousness is lost, remains close to its waking value under ketamine, flattening instead in the high-frequency range in keeping with the drug’s specific action on fast oscillations; this slope tracks the presence of consciousness closely enough to correlate with a perturbational index of cortical complexity [39]. Complexity itself behaves accordingly. The perturbational complexity of the cortical response is preserved at near-waking levels under ketamine even when responsiveness is lost [40], and measures of spontaneous signal complexity show that subanesthetic ketamine raises complexity above the waking baseline while anesthetic doses alternate between lower and higher values before stabilizing at a level comparable to wakefulness [41]. Criticality-based EEG analyses further support ketamine’s divergence from conventional anesthetics: whereas propofol and xenon shift EEG dynamics away from wake-like criticality, ketamine appears to preserve more wake-like dynamical features despite behavioral unresponsiveness [42]. These findings indicate that an active or complex EEG can coexist with ketamine-induced behavioral unresponsiveness. They do not, by themselves, establish anesthetic adequacy, environmental disconnectedness, or the absence of connected awareness.

### 4.4. Why Processed Depth Indices Mislead

These signatures provide a plausible explanation for why processed depth-of-anesthesia indices behave discordantly with ketamine. Many processed indices were developed and validated predominantly under GABAergic-dominant anesthetics, in which deepening hypnosis brings progressive slowing and rising low-frequency power; ketamine instead increases beta and high-gamma (>≈33 Hz) power, and this excess high-frequency activity is the most likely reason the indices may remain elevated or rise despite otherwise stable anesthetic conditions [9]. Clinical studies are consistent with this interpretation. Ketamine increases the bispectral index and spectral entropy even as hypnosis deepens under a volatile agent, an effect that can prompt inappropriate deepening of the primary anesthetic if taken at face value [11]. The magnitude and abruptness of the excursion depend on both the dose and administration pattern, and the apparently conflicting reports are reconcilable once each is specified. Under stable propofol anesthesia titrated to a bispectral index near 40, a 0.5 mg/kg bolus raised the index to a mean of 63.5 whereas 0.2 mg/kg gave a mean of 42.0, indistinguishable from saline [43]; a 0.5 mg/kg bolus produces a comparable rise under sevoflurane [11]. By contrast, 0.2 mg/kg delivered over five minutes left the index unchanged over the following 15 min [44]. In a randomized comparison during major surgery, hourly 0.25 mg/kg boluses produced a greater index increase than a continuous infusion delivering the same hourly dose. A more volatile anesthetic was given in the bolus group to hold the index below 60 [12]. Continuous infusion does not eliminate the effect. During stable sevoflurane anesthesia, esketamine raised the bispectral index dose-dependently: at 0.125 mg/kg/h the index rose by about 5 points at 45 min and 6 at 60 min, whereas at 0.25 mg/kg/h it rose by approximately 11 and 12 points at the same timepoints, with values exceeding 60 in 51% and 54% of patients respectively. A sustained infusion can therefore also generate a clinically relevant processed-index elevation, although it develops more gradually than after a bolus and is specific to the enantiomer, dose, and background anesthetic studied [45]. Racemic ketamine infused at the same hourly rate appears to move the index less [12], but the enantiomers have not been compared directly at matched infusion rates, and the contrast is therefore indirect. The interpretive problem is therefore most acute in the minutes immediately after a bolus. A sustained infusion may nonetheless produce a comparable elevation, developing over a longer interval. A further and clinically consequential limit follows: while ketamine’s structured oscillations and coherence signatures may support monitoring of the anesthetic state and arousal, the available surface-EEG evidence indicates they are not a substrate for monitoring dissociation, which appears to arise from activity that scalp recording does not capture [35]. The practical upshot for both the operating room and the intensive-care unit is that ketamine’s EEG should be read as a qualitative signature of drug effect and arousal—interpreted against the raw tracing and the spectrogram—rather than through a single processed number calibrated to a different class of drug.

## 5. Macroscale Network Dynamics

The local signatures of Section 4 are accompanied by changes in how cortical regions communicate, and it is at this network level that ketamine’s departure from GABAergic anesthesia is most informative—and most easily over-read. Two cautions frame what follows. First, the organizing theme is reorganization rather than suppression: ketamine does not quiet the cortex into a low-activity state, so its network effects are better understood as altered communication within an active brain than as the increased low-frequency synchronization and reduced large-scale connectivity commonly observed with GABAergic-dominant anesthetics. Second, the methods that reveal these effects differ sharply in reach. Much of the richest evidence comes from functional magnetic resonance imaging (fMRI) and intracranial recording in healthy volunteers, psychiatric cohorts, or small surgical series, often at subanesthetic doses—settings far removed from the operating room or intensive-care unit. Only a subset of network measures can presently be derived from a bedside montage, and we flag that distinction throughout.

### 5.1. Cortical Activation and Reduced Network Integration

Ketamine may preserve thalamic activity and cortical metabolism while reducing measures of network integration. In a comparative imaging study, decreased thalamic activity tracked disconnectedness during propofol, dexmedetomidine, and sevoflurane but not during S-ketamine [46]. Yet this heightened activity is accompanied by reduced network integration. In animal work ketamine broadly raises regional metabolism while lowering metabolic connectivity across cortical networks [47], and a rat electroencephalography–microdialysis study found a global reduction in cortical coherence despite increased prefrontal acetylcholine during ketamine-induced unconsciousness [48]. This dissociation between cortical activation and cortical integration is, on the cholinergic side, preclinical evidence; human electroencephalographic studies independently demonstrate disruption of long-range and feedback-directed cortical connectivity [16,49]. The functional significance of the preserved thalamic activity is worth making explicit. The thalamus is not merely a relay but the principal hub sustaining cortical arousal and coordinating cortico-cortical communication; GABAergic anesthetics that deactivate it withdraw this arousal drive, and cortical activity collapses toward the low-complexity, disconnected state of unconsciousness. Together, these findings support a model in which cortical activation and network integration can diverge under ketamine: the cortex may remain active and metabolically engaged while the long-range, feedback-directed communication that binds experience to the external environment is disrupted. Whether preserved thalamic activity causally supports internally generated experience or environmental disconnection remains untested and should be regarded as a mechanistic hypothesis rather than an established explanation. It rests on a single comparative imaging study of thalamic deactivation across agents together with a functional–anatomical argument about the thalamus as an arousal hub, and the causal step—that preserved thalamic drive is what converts unconsciousness into disconnection—has not been tested directly.

### 5.2. Frontoparietal Directional Connectivity as a Candidate Cross-Agent Marker

The most monitoring-relevant network finding concerns the direction of frontoparietal communication, and it is notable precisely because it derives from scalp EEG and converges with the GABAergic agents from which ketamine’s spectral signature otherwise diverges. In the awake state, anterior-to-posterior (“feedback”) influence dominates frontoparietal exchange. Using an information-theoretic measure of directional connectivity, ketamine induction (2 mg/kg) was shown to selectively inhibit feedback connectivity while preserving feedforward connectivity, abolishing the feedback dominance of the conscious baseline—the same pattern produced by propofol and sevoflurane despite their molecular and spectral differences [49]. A complementary analysis confirmed that although ketamine does not reproduce propofol’s frontal-alpha power or alpha-coherence changes, it does reduce both functional and directional frontal-to-parietal connectivity in the alpha band, as propofol does [16]. Together these results suggest that directional, feedback-oriented connectivity may be a candidate marker of altered connectedness or anesthetic state—an EEG-derived measure that may provide complementary information when conventional spectral indices are discordant with clinical state. An important limit accompanies the finding: this work indexes connected (external) consciousness, mediated by lateral frontoparietal networks, rather than the disconnected internal experience that may persist under ketamine [49]—a distinction taken up in Section 6.

### 5.3. Large-Scale Network Reorganization on Functional Imaging

Functional imaging adds resolution the bedside cannot match, at the cost of clinical reach. Resting-state fMRI shows that ketamine produces a network-specific breakdown of functional connectivity rather than a uniform loss, with disruption of frontoparietal and default mode coupling prominent among the changes [17]. A more recent analysis using network control theory characterized the reorganization in dynamical terms: ketamine shifted resting-state dynamics toward increased dominance of a default mode meta-state and decreased dominance of a somatomotor meta-state, a configuration resembling the pretreatment dynamics of patients with post-traumatic stress disorder [50]. This framing was motivated by work on serotonergic psychedelics, which flatten the energetic landscape governing transitions between brain states and render dynamics more entropic [51]; notably, however, the same study found that ketamine did not produce this landscape flattening—the entropy-related indices were unchanged—so the resemblance to psychedelic dynamics holds at the level of network reconfiguration but not at the level of transition energetics. These analyses are conceptually important for understanding dissociation as a reorganization of large-scale dynamics, but they were obtained with fMRI in awake or subanesthetic, frequently non-surgical populations; they speak to mechanism and to the dissociative range rather than to anesthetic-depth monitoring, and none is presently deployable at the bedside.

### 5.4. Intracranial and Information-Theoretic Signatures

**Figure 2:**
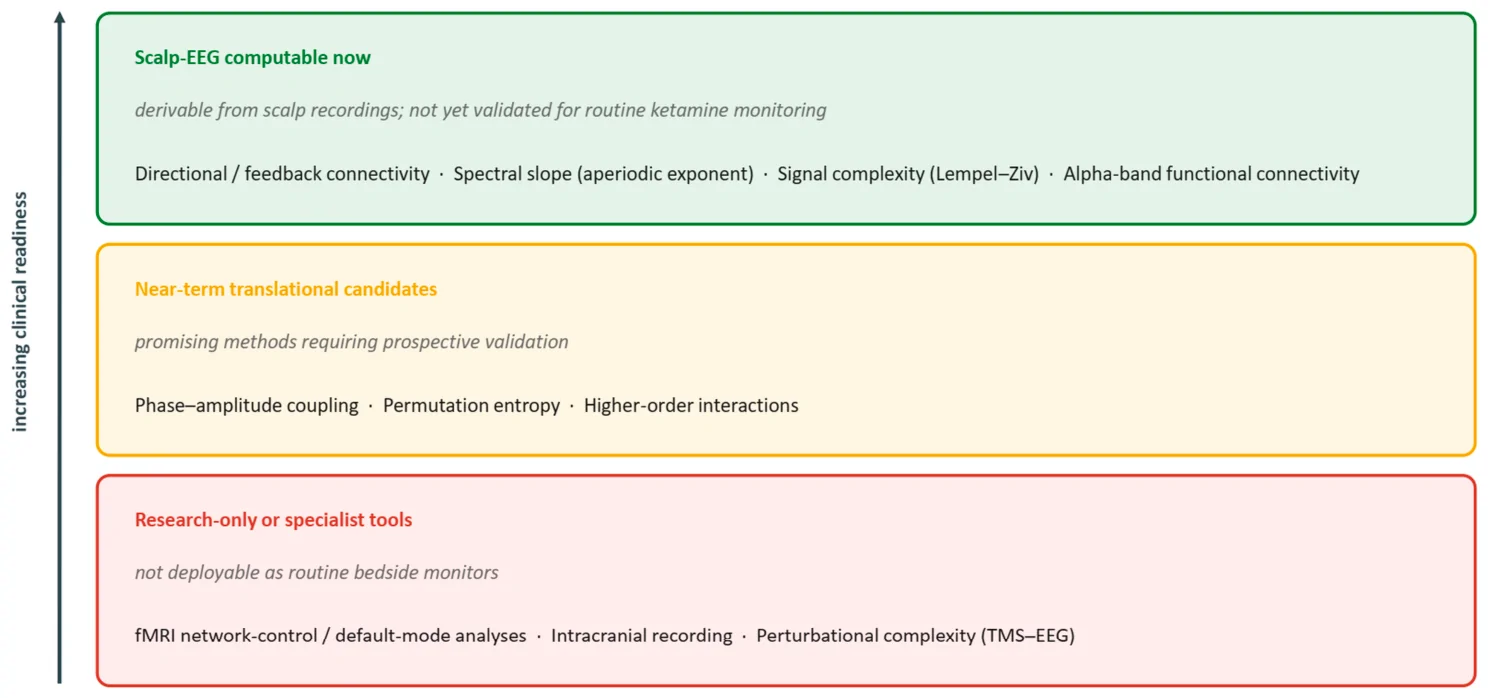
Translational status of ketamine EEG and network measures. Ketamine-related EEG and network measures are grouped by proximity to clinical translation. Scalp-EEG-computable measures can be derived from routine recordings but remain insufficiently validated as ketamine-specific monitoring endpoints. Near-term candidates require prospective validation, whereas research-only tools remain mechanistically informative but are not routine bedside monitors. Abbreviations: EEG, electroencephalography; fMRI, functional magnetic resonance imaging; TMS-EEG, transcranial magnetic stimulation–electroencephalography.

Intracranial recording resolves the activity a frontal montage cannot. In patients implanted for clinical reasons, subanesthetic ketamine engaged distinct regions in distinct frequency bands—gamma activity in the prefrontal cortex and hippocampus alongside a roughly 3 Hz rhythm in the deep posteromedial cortex and attenuation of alpha in the precuneus and temporoparietal junction—a dissociation of spatial and spectral patterns interpreted as separating the circuits underlying ketamine’s antidepressant and dissociative effects [38]. This is the activity alluded to in Section 4.2: relevant to the subjective state but invisible to routine scalp recording. A more translatable development comes from information theory applied to low-density EEG, where ketamine increases redundancy and high-order interactions—most markedly in the alpha band and during rest—in a manner that correlates with derealization and the drift toward dissociative experience [52]. Taken together, the network literature sorts into three tiers by translational status (Figure 2). The first comprises measures already computable from scalp recordings: directional and feedback connectivity, alpha-band functional connectivity, signal complexity, and the spectral slope. None of these is yet validated for routine ketamine monitoring, but each is technically derivable from scalp EEG, although several require multichannel anterior–posterior coverage that routine frontal-only depth monitors do not provide. The second comprises near-term candidates that are technically obtainable from clinical recordings but require prospective validation before clinical use: phase–amplitude coupling, permutation entropy, and the high-order-interaction metrics described above. The third comprises measures that remain research tools—fMRI default mode and network-control analyses, perturbational complexity requiring transcranial magnetic stimulation, and intracranial recording—which carry much of the mechanistic insight but cannot presently be brought to the bedside. Available studies suggest that ketamine’s anesthetized state is marked less by a change in the magnitude of cortical activity than by a change in its functional organization and that this organizational signature is a promising target for future monitoring research.

It is worth stating explicitly which of these constructs a clinical recording can actually deliver, since the distance between the network literature and the bedside is as much methodological as conceptual. Loss of frontoparietal feedback is approximated at the scalp by reduced frontal-to-parietal directed connectivity and by reduced alpha-band coherence between frontal and posterior electrodes; both require at least one posterior electrode, which the frontal-only montages of commercial depth monitors do not provide. Signal complexity and the spectral slope can be computed from a single frontal channel and are therefore the most readily deployable, although both are sensitive to the high-frequency electromyographic artifact that predominates in the lightly anesthetized or unparalyzed patient—the very state in which ketamine is most often given. Phase–amplitude coupling and permutation entropy are computable from clinical recordings but require longer artifact-free epochs than routine monitoring reliably supplies. Measures derived from functional imaging, intracranial recording, or transcranial magnetic stimulation have no scalp-EEG surrogate at all and should be read as mechanistic evidence rather than as candidate monitors.

## 6. Dissociation, Disconnectedness, and Responsiveness

The interpretive difficulties of the preceding sections converge on a conceptual point for which ketamine provides a useful model: the properties clinical practice treats as a single thing—being “under” anesthesia—are in fact separable. Responsiveness, connectedness, and consciousness can come apart, and ketamine frequently separates them. Throughout, we use dissociation for the specific drug-induced state ketamine produces and disconnection (or disconnectedness) for the underlying loss of connectedness to the environment that characterizes it; dissociation is thus one instance of disconnected consciousness.

### 6.1. Three Axes That Usually Move Together

Clinical anesthesia has long used responsiveness—movement or response to command—as a proxy for consciousness, but the two are not equivalent. Consciousness is subjective experience of any kind; connectedness is the specific experience of the external environment; and responsiveness is overt behavioral reaction. These dissociate, and the isolated forearm technique demonstrates that they do: when neuromuscular blockade is prevented from reaching one arm, a substantial minority of apparently anesthetized patients can respond to command. In a meta-analysis of 22 studies and 1131 patients, 393 (34.8%) responded at some point during induction or maintenance, the pooled proportion being 19.7% (95% CI 17.5–22.1) at induction and 31.2% (95% CI 27.8–34.8) during maintenance; heterogeneity between studies was high and only seven were randomized trials, so these estimates should be read as approximate [53]. These findings indicate that connected responsiveness, and potentially connected conscious experience, may persist in a meaningful fraction of patients despite a clinical appearance of unconsciousness [54]. The everyday counterpart is dreaming, in which experience continues while the environment drops away—consciousness without connectedness—and intraoperative dreaming is reported by a substantial minority of unresponsive anesthetized patients [55,56]. On this account, some theoretical frameworks define the clinically relevant goal of general anesthesia as the prevention of connected conscious experience rather than the abolition of all internally generated experience, the experience of surgery, whether by rendering the patient unconscious or by disconnecting an ongoing consciousness from the environment; the proposed mechanism is that subcortical substrates of spontaneous responsiveness are disabled while corticothalamic information integration can continue to generate consciousness and intact noradrenergic signaling sustains connectedness [54]. The functional disconnection of lateral frontoparietal networks that recurs across anesthetic agents is best understood in exactly these terms—as the loss of connected, environment-directed consciousness rather than of consciousness as such [57].

### 6.2. Ketamine as a Model of Disconnected Consciousness

Ketamine frequently produces altered self-environment integration while internally generated experience may persist. The network findings of Section 5 are consistent with this reading: ketamine reduces the anterior-to-posterior frontoparietal connectivity associated with connected, external consciousness [49] even as the cortex remains active and complex. These observations support the use of ketamine as a model of disconnected conscious experience; they do not establish that every unresponsive patient receiving ketamine remains conscious or that a single network mechanism explains dissociation. Consistent with this, ketamine is associated with a high probability of reporting disconnected, dream-like conscious experience on emergence—in one study all participants emerging from ketamine reported long, vivid dreams unrelated to the environment, in contrast to the absence of such reports after propofol or xenon [40]. The dissociative axis appears genuinely distinct rather than a milder grade of anesthesia: ketamine’s dissociative and analgesic effects are independent of one another [58], and intracranial evidence suggests its dissociative and antidepressant effects may be carried by separate circuits [38]. Where deepening a GABAergic anesthetic moves a patient toward unconsciousness, increasing ketamine moves them, characteristically, toward disconnection—an active, internally conscious, environmentally detached state. Preclinical cortical-wave work suggests that ketamine may suppress stimulus-evoked feedback signaling while allowing spontaneous feedback-like activity to emerge, a pattern that could help explain the coexistence of sensory disconnection and internally generated perceptual experience [59].

### 6.3. What This Means for “Depth” and for Monitoring

Two consequences follow, both clinically material. First, unresponsiveness is an especially unreliable proxy for unconsciousness under ketamine: an unresponsive patient receiving ketamine may retain internally generated conscious experience while disconnected from the operating environment, so behavioral stillness supports fewer inferences about experience than it does under agents that act principally by suppression. Second, the EEG markers discussed earlier appear to index specific rungs of this hierarchy rather than consciousness in the abstract. In a systematic evaluation using explicit definitions of responsiveness, connectedness, and consciousness, no proposed resting-state EEG correlate of unresponsiveness was specific to consciousness alone; instead, loss of front-to-back symbolic transfer entropy—the directional-connectivity measure of Section 5—tracked connectedness specifically, while the transition from disconnected consciousness to unconsciousness was marked by falling permutation entropy and a flattening spectral exponent [60]. Behavioral unresponsiveness under ketamine should therefore not be equated with the absence of conscious experience. Available EEG measures may relate to different components of brain state, but they derive from small experimental cohorts and are not validated bedside classifiers. The connected-versus-disconnected distinction is accordingly a clinically relevant research construct rather than a currently measurable monitoring endpoint, and ketamine is among the more useful models for pursuing it.

### 6.4. Ketamine in Context: Other Agents and Dissociative States

Ketamine’s uniqueness is thrown into relief by comparison with other agents that alter consciousness without GABA-A potentiation and with other states in which experience decouples from the environment. Among sedative agents, the α2-adrenergic agonist dexmedetomidine produces a genuinely sleep-like EEG—spindle oscillations and slow waves closely resembling non-REM sleep—by recruiting endogenous sleep pathways, and light dexmedetomidine sedation preserves arousability and can leave connected consciousness intact [61,62]. It presents a contrasting profile: a monitorable, sleep-mimicking pattern rather than an activation-preserving one. More instructive is the comparison within the NMDA-antagonist class itself, which shows that NMDA blockade does not dictate a single signature. Nitrous oxide reduces alpha and delta while raising high-beta activity and, like ketamine, is poorly tracked by the bispectral index [63]; xenon, though also an NMDA antagonist, produces electroencephalographic slowing and a reduction in frontal peak alpha frequency resembling the changes seen with conventional anesthetics—a pattern that, despite shared NMDA antagonism, contrasts sharply with ketamine’s high-frequency activation [64]. That two NMDA antagonists produce nearly opposite electroencephalographic and monitoring behaviors, and that neither matches ketamine’s gamma-burst, indicates that the receptor target alone poorly predicts the surface signature—the specific pharmacology and kinetics matter. Yet at the network level a convergence reappears: nitrous oxide, like ketamine and the volatile agents, reduces frontal and frontoparietal functional connectivity, consistent with the proposal that disruption of long-range, posteriorly directed communication is a possible cross-agent correlate of reduced consciousness even when spectral signatures diverge [49,63]. Finally, ketamine’s state finds its closest neighbors not among the anesthetics but among other dissociative conditions: its large-scale dynamics resemble those of post-traumatic stress disorder [50], and its connectome-level signature aligns with serotonergic psychedelic states and specifically not with propofol-induced or brain-injury unconsciousness [18]. Ketamine-induced states therefore share selected network and phenomenological features with dissociative and psychedelic states, although the extent of this overlap and its relevance to clinical monitoring remain uncertain.

## 7. Clinical Implications for Anesthesia and Critical Care

The signatures and concepts of the preceding sections converge on a single practical message: because ketamine can preserve substantial cortical activity while altering network integration, conventional suppression-based interpretations of brain-state monitoring transfer imperfectly to ketamine-containing regimens, and its EEG must be read qualitatively and in context rather than through a single processed number.

### 7.1. Depth-of-Anesthesia Monitoring and Ketamine as an Adjunct

The most common point of contact is ketamine added to a GABAergic-based anesthetic for analgesia and opioid sparing. In this balanced-anesthesia context, ketamine commonly decreases alpha and low-beta power and increases high-frequency activity, and that excess of beta and gamma is the principal reason processed depth indices rise [9,14]. Under sevoflurane, ketamine increases the bispectral index and spectral entropy even as hypnosis deepens, so a clinician who titrates to the number risks inappropriately deepening the primary agent [11]. The practical response is threefold. An index increase after ketamine should be considered a possible drug-related effect before it is interpreted as light anesthesia. Interpretation should integrate the dose or end-tidal or effect-site concentration of the background hypnotic, the raw EEG and spectrogram, surgical stimulation, neuromuscular-blockade status, and the overall clinical context. Because the index disturbance is largest and most abrupt after boluses, a continuous low-dose infusion may produce a less abrupt excursion than repeated boluses, although infusion can also elevate the index, and the clinician should be most cautious in interpreting the EEG in the minutes following any bolus. When ketamine is the sole or primary anesthetic, the index should not be used as a stand-alone measure of the ketamine-associated anesthetic state, with monitoring falling back on the raw tracing, the spectrogram, and clinical assessment.

### 7.2. Co-Administered Agents and Administration Kinetics

The scenario clinicians meet most often is low-dose ketamine added to propofol-based total intravenous anesthesia, and its EEG interaction is now reasonably well described. When ketamine is given during steady-state propofol, the propofol slow-delta/frontal-alpha pattern acquires added beta–gamma activity and its alpha peak shifts upward by roughly 5 Hz, the composite fading as ketamine is cleared [65,66]. Crucially, this occurs even though ketamine and propofol act additively—or, on neural-field modeling of their shared HCN1 action, infra-additively—on hypnosis. Anesthetic depth may therefore deepen while the bispectral index rises: a clinically common example of the index–state discordance described in Section 4.4 [67].

The magnitude and timing of the effect depend on both the dose and administration pattern. A 0.5 mg/kg bolus raises the index, while 0.2 mg/kg may not [43]. Repeated boluses also raise it more than a continuous infusion delivering the same hourly dose [12], reinforcing the bolus-versus-infusion point of Section 3.

Markers less tied to spectral power—phase–amplitude coupling and directional connectivity—are being examined precisely because they may track depth through this interaction where processed indices fail, although none is yet validated for routine use. Beyond this pairing, ketamine’s interaction with volatile agents and with dexmedetomidine is far less characterized, and mapping the EEG of the balanced anesthetics actually delivered is a clear research priority.

Three co-administered classes deserve specific mention. At usual analgesic doses, opioids generally produce less distinctive independent frontal hypnotic EEG signatures than propofol or volatile anesthetics, so a rising index in a patient receiving ketamine and an opioid should not be attributed to the opioid; their contribution is indirect, reducing hypnotic requirement and so lowering the GABAergic background against which the ketamine signature must be read. Dexmedetomidine produces spindle and slow-wave activity resembling non-rapid-eye-movement sleep rather than the coherent frontal alpha of propofol [61], so combining it with ketamine superimposes a fast, non-suppressive pattern on a sleep-like background; no processed index has been validated against this combination, and the pairing is common in procedural sedation. Volatile agents generate slow and coherent alpha activity broadly similar to propofol, and the randomized desflurane data cited above in [12] provide the clearest demonstration that a ketamine-driven index rise leads clinicians to deepen the volatile background. In each case the practical inference is the same: establish which agent is generating the hypnotic component of the recording and judge depth against that agent rather than against the index.

### 7.3. Reading the Raw EEG and Spectrogram

This places a premium on inspecting the unprocessed signal. At anesthetic doses the recognizable target is the gamma-burst pattern—slow-delta alternating with gamma, with prominent theta—which supports the presence of a ketamine-associated bolus pattern under the conditions studied rather than light anesthesia [9]. The spectrogram makes the high-frequency, non-suppressive character of the state visible at a glance and distinguishes it from the slow-and-alpha pattern of a propofol or volatile anesthetic. Where directional-connectivity analysis is available, the loss of anterior-to-posterior frontoparietal communication offers a state marker that, unlike the spectral indices, behaves consistently across drug classes [49,60]. A realistic appraisal is warranted here; however, directional connectivity is at present a research analysis, not a deployable monitor. The measures are computed offline from multichannel recordings, and no device currently displays them in real time. Bringing them to the bedside would require at least three developments: real-time, artifact-robust computation from the limited montages used clinically; prospective validation against explicit connectedness and consciousness endpoints during maintenance anesthesia and intensive-care sedation, rather than only at induction or in volunteers; and demonstration that the measure tracks ketamine’s state specifically enough to guide titration. Until then it is best regarded as the most promising direction for a ketamine-aware monitor, not a current option.

### 7.4. Sedation and Continuous EEG in Critical Care

Similar considerations apply to analgesia-based sedation in the intensive-care unit, where ketamine is increasingly used and continuous EEG (cEEG) increasingly recorded. Processed sedation indices carry the same liability they carry in the operating room, and depth of sedation is better tracked with a validated clinical scale supplemented by inspection of the raw cEEG than by a single derived value, consistent with current critical-care guidance on the assessment of sedation depth [68,69]. Guidance specific to ketamine analgosedation in mechanically ventilated adults reaches a compatible conclusion from a different direction, judging the supporting evidence to be of low to very low certainty and recommending against ketamine monotherapy where other analgosedative agents are available [70]. The increase in the fast activity ketamine produces should be expected and not mistaken for under-sedation or agitation, and cEEG changes should be interpreted against the patient’s ketamine exposure.

### 7.5. Refractory Status Epilepticus: A Distinct Interpretive Problem

Ketamine’s role in refractory and super-refractory status epilepticus (RSE/SRSE) raises a different and under-appreciated challenge. Its use here is increasingly common and mechanistically rational: as seizures persist, synaptic GABA-A receptors are internalized and GABAergic anesthetics lose potency, while NMDA receptors are trafficked to the synapse, so an NMDA antagonist becomes correspondingly more attractive [71,72]. A 2026 systematic review and meta-analysis of 14 observational studies involving 388 adults estimated a pooled seizure-cessation rate of 64% (95% CI 49–76%), with moderate heterogeneity (I^2^ = 54.1%); ketamine was generally adjunctive and is typically introduced late, alongside propofol or midazolam [13,73]. That estimate should be interpreted cautiously: the contributing studies were observational rather than randomized, and they were heterogeneous in treatment timing, dosing, concomitant therapy, and seizure-cessation definitions, and ketamine’s position late in the treatment sequence confounds attribution to the drug itself. Descriptions of the patterns being treated should follow the standardized critical-care EEG terminology, which defines the ictal–interictal continuum against which any ketamine-associated change must be judged [74]. The interpretive difficulty is that titration in this setting is defined by the EEG itself—by suppression of ictal and interictal-continuum activity, and in some protocols by attainment of burst suppression—yet ketamine’s intrinsic signature is high-frequency and activating, the opposite of the quieting that defines the GABAergic endpoint. Interpretation should therefore focus on the reduction of evolving rhythmic and epileptiform patterns specifically, not by any global quieting of the trace, and avoid two symmetrical errors: assuming that fast activity alone represents persistent or recurrent ictal activity and reading the persistence of fast activity as treatment failure when ictal patterns have in fact resolved. Direct evidence on how ketamine’s oscillatory signature should be distinguished from ictal activity on continuous EEG is sparse—itself a gap worth naming.

### 7.6. Applying the Three-Axis Model at the Bedside

Responsiveness, connectedness, and conscious experience should be considered separately during ketamine administration. Behavioral unresponsiveness alone does not establish the absence of experience or adequate anesthesia. Connectedness—whether the patient is experiencing the operating environment—is the axis that matters most for preventing awareness of surgery and the one the directional-connectivity measures of Section 5 come closest to indexing, although no currently available bedside monitor can reliably distinguish disconnected conscious experience from unconsciousness. When ketamine is used as an adjunct, adequacy of the primary hypnotic should therefore be assessed using its dose or end-tidal or effect-site concentration, the raw EEG and spectrogram, surgical stimulation, neuromuscular-blockade status, and the overall clinical context, rather than a ketamine-inflated processed index. Clinicians should focus on established awareness-prevention strategies and counsel patients about possible dream-like or emergence experiences without escalating the anesthetic solely to normalize the processed index.

### 7.7. Age-Dependent Considerations and Special Populations

Two features of how ketamine is used deserve explicit note, because both stretch the evidence base assembled above. The first is pediatric practice, where ketamine is a mainstay of procedural sedation and a common analgesic adjunct with well-established weight-based dosing [75]. Almost all of the EEG and network characterization reviewed here derives from adults, and the pediatric EEG is not a scaled-down version of the adult: dominant rhythms are slower and mature through infancy, spectral power and its scalp topography shift with myelination and synaptic pruning, and the coherent frontal alpha that anchors depth interpretation in adults is absent in infants under sevoflurane [76]. Processed indices are correspondingly less reliable in infants and young children: in a prospective study of 50 children aged 4 to 24 months, processed indices showed unexpectedly elevated values during otherwise stable anesthesia maintenance in 70% of patients [77], which further limits direct extrapolation of adult ketamine-monitoring patterns to this population. Adult dose–signature relationships therefore cannot be assumed to transfer, and no dedicated study has defined pediatric ketamine EEG patterns across the dose–state continuum. Prophylactic esketamine has been reported to reduce rather than provoke sevoflurane-associated emergence delirium in children [78], though this does not establish an age-specific monitoring signature. Large pediatric anesthesia studies are broadly reassuring after a single brief exposure [79,80], while cohort data after repeated exposure indicate more subtle decrements in processing speed and fine motor function [81]; these studies concern general anesthetic exposure rather than ketamine specifically, and none resolves the effect of prolonged ketamine administration.

The second feature is repeated and prolonged exposure. The signatures central to this review derive overwhelmingly from single doses, yet ketamine is frequently given repeatedly—as serial infusions or maintenance esketamine for depression and as prolonged infusions for intensive-care sedation and status epilepticus. Whether the acute signature persists, attenuates, or adapts across repeated or continuous exposure is uncharacterized. Receptor adaptation or tolerance could plausibly alter the disinhibition–AMPA balance that generates it, and cumulative network effects could diverge from the single-dose picture, but neither has been demonstrated electrophysiologically and both should be regarded as research hypotheses. Because these are precisely the settings in which clinicians most often rely on the EEG, the absence of validated guidance for these populations and regimens is a clinical void rather than a theoretical one.

The same caution applies at the other end of life. Electroencephalographic power falls with age and the coherent frontal alpha that anchors depth interpretation under propofol and sevoflurane weakens progressively, so an older patient at a given anesthetic concentration shows a pattern that in a younger patient would suggest greater depth and is correspondingly more susceptible to burst suppression [82]. Two consequences follow for ketamine. A ketamine-driven index rise superimposed on an age-attenuated background may be read as adequate wakefulness when the GABAergic contribution is in fact excessive, and the raw-EEG anchors recommended throughout this review—frontal alpha, slow-wave amplitude—are themselves diminished in the frail older patient, so the fallback on which the whole interpretive strategy depends is weakest precisely where it is most needed. No study has characterized ketamine’s EEG signature specifically in older or frail patients. This should be treated as an evidence gap rather than as a settled extrapolation, and it is recorded as such in the evidence table presented in Section 9.

### 7.8. A Practical Stepwise Interpretation Framework

**Figure 3:**
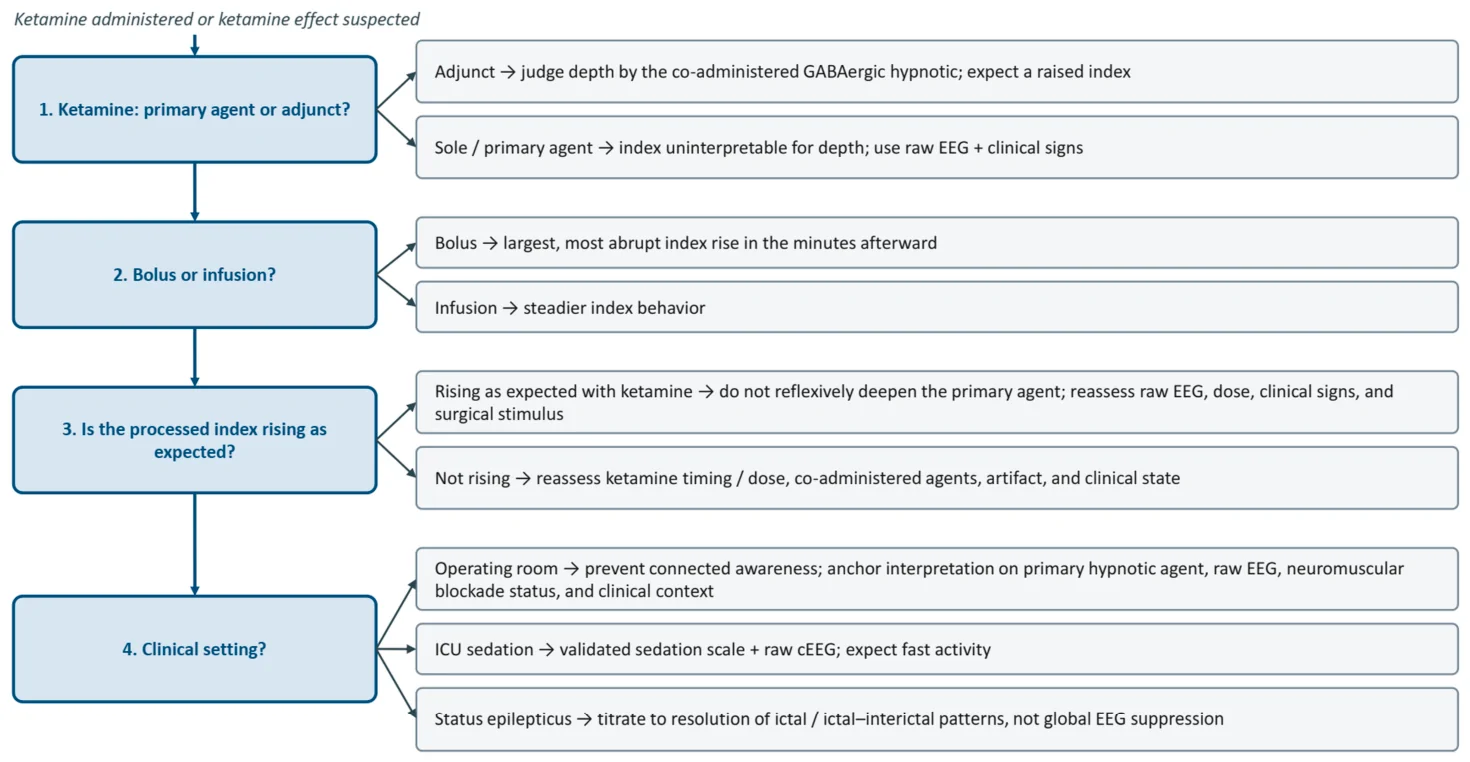
Decision aid for interpreting EEG during ketamine administration. The decision aid summarizes a pragmatic approach to EEG interpretation when ketamine is used as an adjunct or primary anesthetic/sedative agent. Processed indices are not validated as stand-alone measures of the ketamine-associated anesthetic state, and a rising index should not automatically be interpreted as light anesthesia; raw EEG, spectrogram features, dose, co-administered agents, clinical signs, neuromuscular blockade, and clinical context should be integrated. In status epilepticus, titration should focus on resolution of ictal or ictal–interictal continuum patterns rather than global EEG suppression. Abbreviations: cEEG, continuous electroencephalography; EEG, electroencephalography; GABA, gamma-aminobutyric acid; ICU, intensive-care unit.

The foregoing reduces to a short interpretive sequence, summarized as a decision aid in Figure 3:

1. Identify the regimen and administration pattern. Establish whether ketamine is the primary anesthetic, a balanced-anesthesia adjunct, a sedative, or an antiseizure agent, and whether it is given by bolus or infusion, since each changes what the EEG and any index mean [12,43,45]. When ketamine is the sole or primary agent, the processed index should not be used as the sole measure of anesthetic state; when it is an adjunct, depth remains anchored to the co-administered hypnotic.
2. Distrust the single number. If the index rises after ketamine administration, consider a ketamine-associated index effect [43] before interpreting the change as light anesthesia and do not deepen a co-administered hypnotic on the index alone [12]. If the index is high during a steady-state infusion, the same caution applies, though the rise develops more slowly and may be missed when only intermittent values are recorded.
3. Read the raw EEG and spectrogram. If a gamma-burst or an alternating slow-gamma pattern is present, anchor depth on the co-administered GABAergic agent rather than on the index, since the pattern supports ketamine effect under compatible bolus conditions but does not establish anesthetic adequacy [8,14]. If high-frequency activity persists while the patient is clinically dissociated, do not escalate the volatile or propofol concentration in response to the index alone. Absence of this pattern does not exclude ketamine effect.
4. Anchor interpretation to the background hypnotic and clinical context. Integrate the dose, end-tidal, or effect-site concentration of the co-administered hypnotic, the raw EEG and spectrogram, surgical stimulus, neuromuscular-blockade status, movement where assessable, and the overall clinical context. Hemodynamic and autonomic responses may be supportive observations but are not primary measures of anesthetic adequacy.
5. In status epilepticus, define the endpoint by ictal-pattern resolution. Titrate to suppression of seizure and interictal-continuum activity, described using standardized critical-care terminology [13,74], not to overall EEG quieting, and do not confuse ketamine’s fast activity with ongoing seizure.
6. Remember disconnection. Behavioral stillness under ketamine does not establish unconsciousness; behavioral unresponsiveness does not establish absence of conscious experience; internally generated experience may persist [54]; and current monitors do not reliably classify connectedness.

These steps are expert-informed interpretive heuristics rather than validated guidance. They are extrapolated from an evidence base drawn largely from induction and bolus administration rather than from maintenance-phase anesthesia or prolonged infusion, several of the underlying observations come from small single-center studies, and no prospective trial has tested whether following this sequence improves patient outcomes. The sequence is offered to structure interpretation at the bedside, not to substitute for clinical judgment or institutional protocol; the specific uncertainties are set out in Section 9.

## 8. Beyond Anesthesia: Translational Links to Antidepressant Mechanisms

Ketamine’s second major identity—as a rapid-acting antidepressant, and in its S-enantiomer an approved treatment for treatment-resistant depression—is usually treated as separate literature. It is better read as the same network story viewed through a therapeutic lens, because the antidepressant effect may share an upstream cortical event with the anesthetic signatures of Section 4 and Section 5: NMDA blockade on GABAergic interneurons, disinhibition of pyramidal cells, and a surge of glutamate.

### 8.1. Potentially Shared Upstream Mechanisms

The glutamate surge that follows interneuron disinhibition activates AMPA receptors, and it is AMPA throughput—rather than NMDA blockade as such—that appears to carry the downstream antidepressant cascade: activation of BDNF–TrkB and mTORC1 signaling, increased synaptic-protein synthesis, and rapid synaptogenesis in the prefrontal cortex and hippocampus that reverses the dendritic-spine loss of chronic stress [83,84,85]. In rodent models the causal weighting favors this pathway: the AMPA antagonist NBQX prevents ketamine’s molecular and behavioral effects, and mTORC1 inhibition attenuates the synaptogenic and behavioral response [83]. This should not be extrapolated uncritically to humans. In a randomized crossover trial in depressed patients, systemic rapamycin pretreatment did not block ketamine’s antidepressant effect at 24 h and appeared to prolong it, which leaves the clinical role of the pathway uncertain [86]. This is the same shift toward AMPA-over-NMDA throughput invoked in Section 2 to explain the increased gamma activity of ketamine’s EEG, so the signature that confounds depth monitors and the cascade that carries the antidepressant response appear to share an upstream molecular trigger. Participant-level analyses support the separability of these components directly. When the same subanesthetic infusion is given under general anesthesia rather than to an awake participant, the high-frequency beta–gamma modulation persists while the characteristic theta augmentation is absent, so the two components can be dissociated by removing conscious awareness rather than by changing the dose [87]. What is shared, on present evidence, is that trigger rather than a common electrophysiological state: the gamma increase is an acute, concentration-dependent effect measurable within minutes of administration, whereas the synaptogenic changes that carry the antidepressant response unfold over hours to days. No study has yet shown the two to be expressions of a single electrophysiological event.

### 8.2. Post-Treatment Network Changes in Depression

At the network level ketamine acts on the same large-scale systems that organize the anesthetic story—default mode, salience, and central-executive networks—but in the direction of normalizing the connectivity abnormalities of depression rather than producing unconsciousness. Resting-state fMRI shows ketamine reducing the default-mode connectivity abnormalities reported in major depression toward healthy-range values, producing changes in prefrontal–limbic coupling, and improving global brain connectivity, with default mode–insula connectivity normalizing around the peak of antidepressant response at day 2 and reverting by day 10 as the effect wanes [88,89]. Two features distinguish this from the anesthetic state: the changes that track clinical response are measured hours to days after infusion, well outside the acute dissociative window, and they are studied almost entirely with fMRI, so they share the translational limitation flagged in Section 5—mechanistically informative, but not bedside measures.

### 8.3. Dissociation and Candidate EEG Biomarkers

Whether the acute dissociation contributes to the antidepressant effect or is a concurrent side effect remains unresolved and bears directly on the framework of Section 6. Some studies report that dissociative intensity predicts subsequent response [90], but current evidence increasingly supports the view that dissociation is at most a correlate or biomarker rather than the therapeutic mechanism, a shift indicated by the independence of ketamine’s dissociative and analgesic actions [58] and intracranial evidence that its dissociative and antidepressant effects engage separable circuits [38] and by the conclusion of a systematic review that dissociation is not necessary for antidepressant efficacy [91]. Recent precision-medicine reviews reach a similar conclusion, suggesting that EEG changes—including frontoparietal gamma power—may be more promising markers of response than dissociative symptoms alone [92]. The question is not fully settled, however, because most biomarker measurements are made hours to days after infusion, well past the acute dissociative window, and subtler associations remain difficult to exclude. The link to the monitoring thesis is concrete rather than thematic: post-infusion gamma power—changes within a related high-frequency spectral domain—has been related to antidepressant response. Recent real-world EEG data in bipolar depression extend these findings to clinical populations, showing reduced low-frequency power, increased gamma power, a flattened spectral slope, and increased entropy after subanesthetic ketamine [93]. Newer work in late-life treatment-resistant depression further suggests that ketamine-induced high-order EEG interactions may evolve over hours to days and may track both dissociative states and clinical response, although these metrics remain exploratory [94]. Measures drawn from the same electrophysiological family are thus a nuisance to be recognized in one setting and a candidate treatment biomarker in another. The parallel is one of measurement rather than of an identical brain state, since the two studies record at different doses, in different populations, and on different timescales. Related electrophysiological measures may ultimately have different interpretive roles in anesthesia and in psychiatry, but their dose-, state-, and time-specific validity requires separate prospective evaluation.

## 9. Knowledge Gaps and Future Directions

The preceding synthesis rests on literature that is, in several respects, thinner and less even than ketamine’s clinical use would warrant. It is useful to state plainly which claims are robust and which provisional. The gamma-burst pattern and ketamine-associated processed-index elevation have been reproduced across several studies, although the relevant human cohorts remain small and administration conditions vary and, for the signature, across species. The spectral-slope, complexity, and frontoparietal directional-connectivity findings are consistent in direction but rest on small samples obtained largely at induction or in volunteers rather than during sustained maintenance anesthesia. The antidepressant network-normalization findings are convergent but fMRI-based, and the characterization of dissociation as a discrete network state relies on very small human samples. The gaps that follow are grouped by whether they limit what can be claimed about the electrophysiology or what can be done with it at the bedside. The evidence underlying each of the principal clinical claims made in this review, together with the main limitation of each evidence base, is set out in Table 3.

### 9.1. Gaps in the Evidence Base

The most basic limitation is the unevenness emphasized throughout: the anesthetic-dose alternating slow-delta and gamma pattern is the best described of the ketamine signatures, though the human cohorts remain small, whereas the oscillatory and network correlates of the subanesthetic and dissociative ranges are sparser and more variable, and the transitions between states remain sparsely characterized. Establishing a validated dose–signature mapping would require dose-controlled human studies that sample the full range within subjects, which remain scarce. Three confounds compound the problem: the administration pattern is entangled with dose across the literature; the racemic and S-enantiomer forms are often treated interchangeably despite pharmacological differences; and ketamine’s interaction with propofol and volatile agents—the combinations that dominate practice—has been characterized only piecemeal. Underlying all three is a reliance on small surgical series, healthy-volunteer imaging, and cross-species inference that constrains how confidently any signature generalizes to the perioperative or critically ill patient, and, as Section 7.7 noted, to children and to repeated exposure.

### 9.2. Gaps in Monitoring Tools

There is at present no validated, dose- or state-specific EEG biomarker of ketamine effect that a monitor could display, and the processed indices in routine use may be discordant with clinical state and can be misleading when interpreted without a ketamine-specific context. Promising candidate measures include directional connectivity and signal complexity, which behave more consistently across drug classes and map onto specific levels of the connectedness hierarchy [49,60], but these are computed offline in research settings, are not yet available as real-time, bedside-robust outputs, and turning them into monitors usable in the operating room and intensive-care unit is a central practical need. A more ambitious target is a marker of disconnection—one that could distinguish an internally conscious but environmentally disconnected brain from an unconscious one—which does not yet exist. The status epilepticus setting needs its own dedicated work on distinguishing ketamine’s activating signature from ictal activity on continuous EEG.

### 9.3. Priorities

Not all of these gaps are equally tractable or consequential, and it is worth ranking them. A high priority, because it is necessary for clinical translation and is directly addressable, is the development and prospective validation of a bedside-deployable EEG marker—most plausibly directional connectivity or a complexity measure—against explicit connectedness and consciousness endpoints during maintenance anesthesia and intensive-care sedation. Second is the dose-controlled characterization of the subanesthetic-to-anesthetic transition within subjects, disentangling dose from administration pattern, which would convert the organizing heuristic of this review into an evidence-based mapping. Third, and specific to a high-stakes population, is the interpretation of continuous EEG under ketamine in status epilepticus. Lower in priority, though valuable, are the finer mechanistic questions—the enantiomer comparison and the precise circuitry separating dissociation from antidepressant action—scientifically important but less rate-limiting for practice. Framing the agenda this way distinguishes what is merely unknown from what, if known, would change what clinicians do.

Two further priorities follow from the variability emphasized throughout. The first is individualized interpretation. The variance in ketamine’s electroencephalographic response is wide and arises partly from characteristics observable before administration—age, prior exposure and tolerance, psychiatric comorbidity, and baseline network organization—which makes population-derived index thresholds particularly ill-suited to this drug. A more tractable target than a universal threshold is within-patient change from a pre-drug baseline recording, and adaptive algorithms that reference a patient’s own EEG rather than a population norm are a plausible direction; none has been validated for ketamine, and none should be presented as presently available. The second is methodological convergence. Progress on the dissociative range in particular will require studies that report standardized frontal and parieto-occipital montages, harmonized theta and gamma metrics with explicit band edges and reference schemes, and effect-site-targeted or infusion-matched dosing so that the administration pattern and rate can be distinguished from dose. Cross-modal designs pairing EEG with functional imaging or intracranial recording in the same subjects under the same dose conditions would do more than any single new modality to anchor the network claims of Section 5 to signals a bedside monitor could compute.

## 10. Conclusions

Ketamine produces dose- and context-dependent EEG patterns that differ from those commonly observed with GABAergic-dominant anesthetics. The best-described anesthetic-dose pattern is alternating slow-delta and gamma activity following bolus administration, whereas lower-dose spectral and connectivity findings are more heterogeneous. Processed indices may remain elevated or increase when ketamine is administered alone or added to another anesthetic; they should therefore not be interpreted without consideration of the raw EEG, spectrogram, administration pattern, background hypnotic, and clinical state.

Human and preclinical studies also suggest that ketamine can preserve substantial cortical activity while disrupting long-range and feedback-directed connectivity, but these network measures are not yet validated for real-time clinical monitoring. Distinguishing responsiveness, connectedness, and conscious experience provides a useful conceptual framework, although no currently available bedside monitor reliably classifies these states. Future studies should prospectively characterize dose- and infusion-dependent EEG transitions, mixed-agent regimens, age-specific effects, and candidate connectivity or complexity measures during maintenance anesthesia and critical care. Potential links to antidepressant action are best understood as shared upstream mechanisms rather than a common EEG state.

## Figures and Tables

**Table 1:** The ketamine dose–state continuum as a practical heuristic, mapping approximate dose ranges to the representative administration pattern, clinical effect, characteristic EEG signature, and processed-index behavior and a corresponding interpretive consideration.

Dose Range	Administration Pattern	Clinical Effect	Key EEG Signature	Processed-Index Behavior	Interpretive Consideration
0.1–0.5 mg/kg	Slow infusion	Analgesia; mild dissociation; antidepressant effect	Reduced alpha and low-beta power	May rise modestly	Clinical scale plus raw EEG
0.5–1 mg/kg	Bolus or infusion	Dissociation; procedural sedation in some regimens	Mixed theta and gamma; reduced alpha	May remain elevated or rise	Use clinical state and raw EEG/spectrogram where available
1–2 mg/kg	Rapid bolus (induction)	Surgical anesthesia (unresponsiveness)	Gamma-burst: alternating slow-delta and gamma	Not validated as a stand-alone depth index	Consider ketamine-associated bolus pattern; interpret against background hypnotic
Emergence/recovery (dose not applicable)	Any	Emergence phenomena; vivid dreams	Variable; often a shift toward alpha-range and slower activity as ketamine is cleared	Lags the clinical state	Clinical awareness; counsel the patient

Doses are indicative and vary with the individual, the enantiomer, and—importantly—the pattern and rate of administration; the table is a heuristic for organizing the evidence, not a validated dose–signature correspondence. The interpretive consideration column likewise summarizes expert-informed interpretation rather than validated clinical recommendation and should be read together with the uncertainties set out in Section 9. EEG, electroencephalography.

**Table 2:** Glossary of the electroencephalographic terms used in this review with the computation each denotes and its approximate frequency range.

Term	Definition and How It Is Computed	Approximate Frequency Range
Gamma-burst	Alternating epochs of high-amplitude slow activity and high-frequency oscillation seen at anesthetic doses; identified on the spectrogram or by tracking slow- and gamma-band power envelopes.	Slow-delta component 0.1–4 Hz and gamma component 27–40 Hz as reported in the canonical human study; broader gamma definitions are used elsewhere
Slow-gamma alternation	The same phenomenon named by its two constituent rhythms; quantified as the periodic anticorrelation between slow-band and gamma-band power.	Slow-delta 0.1–4 Hz; gamma 27–40 Hz
Preserved or wake-like complexity	Preservation of signal irregularity approaching the awake state, in contrast to the reduction seen under GABAergic anesthesia; indexed by complexity or entropy measures.	Broadband, typically 0.5–45 Hz
Spectral slope (aperiodic exponent)	Exponent of the 1/f component of the power spectrum after periodic peaks are separated out; a flatter (less negative) slope indicates a shift toward cortical excitation.	Fitted across ~1–40 Hz
Lempel–Ziv complexity	Compression-based measure of the diversity of binarized signal patterns, normalized against phase-shuffled surrogates of the same spectrum.	Broadband
Permutation entropy	Entropy of the distribution of ordinal patterns within short embedded segments of the signal.	Broadband; band-limited variants are common
Directed (feedback) connectivity	Asymmetry of information flow between regions, estimated using symbolic transfer entropy, Granger-type analyses, or directed phase-based methods; frontal-to-parietal flow is the feedback direction.	Most often alpha, 8–13 Hz
Phase–amplitude coupling	Modulation of the amplitude of a faster rhythm by the phase of a slower one, quantified as a modulation index.	Slow phase 0.5–4 Hz; fast amplitude 8–13 or 30–80 Hz

Frequency boundaries are indicative and vary between studies; where a measure is broadband, the analysis bandwidth typical of the source literature is given. GABA, gamma-aminobutyric acid.

**Table 3:** Evidence underlying the principal clinical claims of this review, with study-level descriptors, the main limitation of each evidence base, and a narrative characterization of the evidence.

Claim	Evidence Base, Regimen, and Principal Limitations	Principal Finding	Evidence Characterization
A ketamine bolus raises the processed index during GABAergic anesthesia	Randomized trial, n = 45 adults, propofol anesthesia; 0.5 mg/kg bolus [43]. Single-center, brief observation, index-only outcome.	Mean bispectral index 63.5, against 42.0 after 0.2 mg/kg and no change after saline	Direct but limited human evidence
The excursion is dose-dependent	The same trial [43], with a randomized sevoflurane comparison, n = 22 [11]; 0.5 versus 0.2 mg/kg. Two studies only, over a narrow range of doses.	0.2 mg/kg was indistinguishable from saline; the effect at 0.5 mg/kg held under sevoflurane	Limited but concordant human evidence
Low-dose slow administration need not raise the index	Randomized double-blind trial, n = 30 (14 ketamine, 16 placebo), propofol–remifentanil; 0.2 mg/kg over 5 min [44]. One dose and one rate; a negative result.	No change over the following 15 min	Direct but limited human evidence
Administration pattern modifies the size of the excursion	Randomized trial, n = 50 adults, desflurane; 0.25 mg/kg hourly bolus versus 0.25 mg/kg/h infusion [12]. Single-center, one dose, volatile background only.	Bolus raised the index, and more desflurane was needed to hold it below 60	Direct but limited human evidence
Sustained infusion can also raise the index	Randomized double-blind trial, n = 120 randomized and 103 analyzed, sevoflurane; esketamine 0.125 and 0.25 mg/kg/h [45]. Esketamine only, one background agent, gradual onset, 60 min observation.	Index rose by about 11 points at 45 min and 12 points at 60 min	Direct but limited human evidence
Anesthetic-dose ketamine produces a gamma-burst pattern	Retrospective human EEG study, n = 12, following anesthetic bolus administration (mean 2.2 mg/kg) [14], with supporting characterization from a clinical EEG review [8]. Small cohort; heterogeneous recording and administration conditions.	Alternating slow-delta and gamma activity, without progression to suppression	Direct but limited human evidence
Subanesthetic correlates are less consistent	Human EEG studies, n = 15 [15] and n = 21 [36], using differing montages and metrics at subanesthetic exposure (0.1–0.5 mg/kg). Heterogeneous methods; no harmonized metrics or montages.	Increased fast activity, with inconsistent band-specific and connectivity effects	Heterogeneous human evidence
Frontoparietal feedback connectivity is disrupted	Human EEG studies from the same author group: n = 30 surgical patients receiving 2 mg/kg for induction [49] and n = 28 in a subsequent analysis [16]. Probably not independent replications; estimates depend on analytic choice and montage.	Reduced frontal-to-parietal directed connectivity, shared with propofol and sevoflurane	Limited human evidence
Cortical activation dissociates from cortical integration	Rat EEG–microdialysis, 11 animals [48], and animal metabolic imaging [47]; anesthetic doses. Preclinical; species, dosing, and recording differences limit transfer.	Globally reduced cortical coherence despite raised prefrontal acetylcholine	Preclinical mechanistic evidence
Connected consciousness persists in a minority of patients	Meta-analysis of 22 studies, n = 1131 patients; various regimens [53]. High heterogeneity; only seven randomized trials; not specific to ketamine.	393 of 1131 (34.8%) responded; 31.2% (95% CI 27.8–34.8) during maintenance	Heterogeneous human evidence; not ketamine-specific
Ketamine has been associated with seizure termination in refractory and super-refractory status epilepticus	Systematic review and meta-analysis of 14 observational studies involving 388 adults [73], with a retrospective (S)-ketamine cohort of n = 42 [13]. No randomized trials; heterogeneous treatment timing, dosing, concomitant agents, and cessation definitions.	Pooled seizure-cessation rate 64% (95% CI 49–76%); moderate heterogeneity (I^2^ = 54.1%)	Limited, heterogeneous observational evidence
Ketamine EEG signatures in children and in frail older adults	No dedicated studies identified; age-specific signatures have not been characterized under ketamine.	—	Direct evidence lacking

Evidence characterizations are narrative judgments based on study design, replication, consistency, directness, and clinical applicability; they do not represent formal GRADE assessments, and no formal risk-of-bias instrument was applied. Bracketed numbers refer to the reference list. CI, confidence interval; EEG, electroencephalography.

## Data Availability

No new data were created or analyzed in this study. Data sharing is not applicable to this article.
